# Factors Associated with Treatment Prescription to Pulmonary Tuberculosis Contacts in Catalonia (2019–2021): A Population-Based Epidemiological Study

**DOI:** 10.3390/vaccines11121800

**Published:** 2023-12-01

**Authors:** Ángela Domínguez, Núria Soldevila, Diana Toledo, Ignasi Parrón, Joan-Pau Millet, Irene Barrabeig, Pere Godoy, on behalf of Transmission of Tuberculosis in Catalonia (Spain) Working Group

**Affiliations:** 1 Departament de Medicina, Universitat de Barcelona, 08036 Barcelona, Spain; angela.dominguez@ub.edu (Á.D.); nsoldevila@ub.edu (N.S.); 2 CIBER de Epidemiología y Salud Pública (CIBERESP), 28029 Madrid, Spain; jmillet@aspb.cat (J.-P.M.); pere.godoy@gencat.cat (P.G.); 3 Agència de Salut Publica de Catalunya, 08005 Barcelona, Spain; iparron@gencat.cat; 4 Agència de Salut Pública de Barcelona, 08023 Barcelona, Spain; 5 Institut de Recerca Biomédica de Lleida (IRBLleida), 25198 Lleida, Spain

**Keywords:** contact tracing, tuberculosis, latent TB infection, treatment, prescription, prevention

## Abstract

In countries with low tuberculosis (TB) incidence, the systematic testing and treatment of latent TB infection (LTBI) in contacts of pulmonary TB index cases is the standard of care. The objective of this study, conducted in Catalonia over 2019–2021, was to assess the factors associated with LTBI treatment prescription to close contacts of pulmonary TB index cases. In this population-based epidemiological study of LTBI prevalence among pulmonary TB contacts between 2019 and 2021, multiple logistic backward stepwise regression was used to identify the factors associated with treatment prescription, for which the adjusted odds ratio (aOR) and 95% confidence intervals (CI) were calculated. A total of 1487 LTBI contacts of 542 pulmonary TB index cases were studied, 80.6% of whom received a prescription. The factors associated with LTBI treatment prescription were exposure ≥6 h/day (aOR 14.20; 95% CI 5.22–38.66) and exposure <6 h/day (aOR 7.32, 95% CI 2.48–21.64), whereas the factors associated with no LTBI treatment prescription were age ≥55 years (aOR 0.22, 95% CI 0.08–0.64) and bacillus Calmette–Guerin vaccination (aOR 0.38, 95% CI 0.16–0.90). Crucial to LTBI treatment prescription is information on the contact’s duration of exposure to pulmonary TB, not only for contacts exposed for ≥6 h/day, but also for contacts with lower daily exposure levels.

## 1. Introduction

Tuberculosis (TB) is a major cause of morbidity and death worldwide. According to a 2022 World Health Organization (WHO) report, TB deaths in 2021 were 1.4 million and 187,000 among human immunodeficiency virus (HIV)-negative and HIV-positive individuals, respectively [1]. The WHO End TB Strategy aim is to achieve a 50% reduction in TB incidence by 2025 [2].

Latent TB infection (LTBI) is when, in the absence of active TB, a persistent immune response to *Mycobacterium tuberculosis* antigens can be demonstrated. Of individuals with LTBI, approximately 10% will develop active TB, half within the early years following infection [3,4]. The fact that one quarter of the world’s population is estimated to have LTBI [5] represents an important reservoir for future active TB cases, especially in risk groups such as the elderly, children, and HIV-positive and other immunosuppressed patients.

Adult and child contacts of pulmonary TB cases are systematically tested and treated for LTBI in countries with low TB incidence [6,7,8], and the recommendation is that treatment should be initiated and completed by at least 85% and 75% of infected contacts, respectively [9]. Nonetheless, the number of household contacts diagnosed with LTBI who are prescribed preventive treatment remains low [1]. Identifying and treating individuals with LTBI is crucial to preventing active TB development and transmission [10].

In Catalonia, a region in northeast Spain with 7.8 million inhabitants, the annual active TB incidence rates per 100,000 inhabitants were 14.1 in 2019 [11], 10.7 in 2020 [12], and 12.5 in 2021 [13], and the corresponding pulmonary disease rates were 64.4%, 66.6%, and 69.8%, respectively. According to the recommendations of the Generalitat of Catalonia’s Department of Health, the tracing of pulmonary TB index case contacts should aim to reduce morbidity and mortality among new cases of TB, detect sources of infection to reduce *M. tuberculosis* transmission, and contribute to TB elimination by treating new infections [14].

The aim of our study was to identify the factors associated with LTBI treatment prescription to close contacts of pulmonary TB index cases in Catalonia over a 30-month period in 2019–2021.

## 2. Materials and Methods

We performed a population-based epidemiological study of LTBI prevalence for all contacts of pulmonary TB cases in Catalonia between 2019 and 2021. The study population consisted of contacts of all new active pulmonary TB cases registered by the Catalan Epidemiological Surveillance Network (attached to the Public Health Agency of Catalonia) in the 30-month period from 1 January 2019 to 30 June 2021. Included in the study were contacts (household, school, workplace, recreational and other indoor settings) of pulmonary TB patients resident in Catalonia. The infectiousness period of the index case was determined to identify contacts, who were considered infected if they had a positive interferon gamma release assay (IGRA) or a tuberculin skin test (TST) induration diameter of ≥5 mm. Contacts underwent a clinical examination and posterior-anterior chest X-ray to rule out active TB.

Catalan Epidemiological Surveillance Network staff carried out an epidemiological survey of cases of pulmonary TB and enlisted household and community contacts by interviewing TB patients and healthcare workers who had reported active TB cases. Contacts were evaluated as soon as possible after index cases were diagnosed, especially if they showed sputum-smear positivity, and within 10–12 weeks after the last index case contact during the infectiousness period.

Registered contacts without active TB were administered the Catalan Epidemiological Surveillance Network [14] contact study questionnaire which was adapted to meet the objectives of the study. After evaluation in December 2018 in a pilot study of 50 contacts, it was considered that no additional changes to the questionnaire were necessary. The questionnaire compiled information on age, sex, migrant status, exposure setting (household, workplace, school, recreational and other), exposure duration (≥6 h/day, <6 h/day, non-daily, sporadic), tobacco use (smoker, ex-smoker, non-smoker), alcohol use, a history of bacillus Calmette–Guerin (BCG) vaccination, HIV, or immunosuppression, and TST or IGRA results. The information collected for pulmonary TB index cases of the studied contacts was as follows: sex, age, migrant status, chest X-ray results, bacteriology results, social vulnerability, HIV infection, tobacco use, alcohol use, and injecting-drug use (IDU). For contacts aged ≤13 years, the tobacco and alcohol variables were marked ‘no’ when data were missing.

Because Catalan treatment prescription guidelines [14] distinguish between contacts aged 0–5 and ≥6 years, we analyzed data overall and also separately for those two age brackets. The odds ratio (OR) and 95% confidence interval (CI) values were calculated to determine the associations between the dependent variable (treatment prescription) and the independent variables. To detect factors independently associated with treatment prescription, multiple logistic regression, performed using the backward stepwise method, included sex and all variables with *p* < 0.2, and the adjusted OR (aOR) and 95% CI values were calculated.

## 3. Results

### 3.1. Characteristics of Infected Contacts

**Figure 1:**
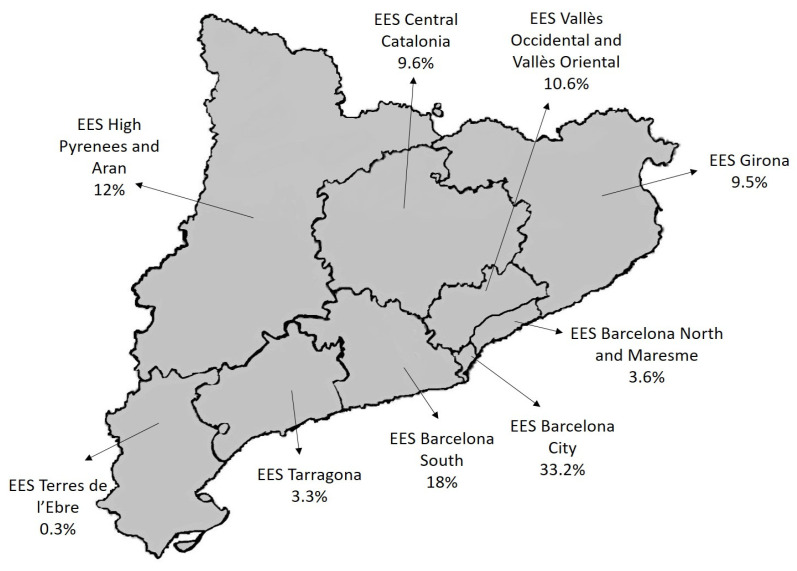
Geographical distribution of the infected contacts.

A total of 1487 infected contacts of 542 pulmonary TB cases were studied. Figure 1 shows the geographical distribution of the infected contacts according to the Catalonia Epidemiological Surveillance Service (ESS) to which they belong. Most of the infected contacts (33.2%) belonged to the ESS of Barcelona City.

Of the 1487 contacts, 642 were migrants and 443 had received the BCG vaccination. Only 64% of the vaccinated contacts had been tested for IGRA. LTBI treatment was prescribed to 80.6% of contacts, with no differences observed by sex (80.6% male and 80.6% female), neither overall nor considering only contacts aged ≥6 years (79.9% male and 79.8% female).

Table 1, which reports bivariate analysis results for contacts, shows that LTBI treatment was more frequently prescribed to contacts aged 0–5 years (OR 3.86; 95% CI 1.17–12.70) and 6–17 years (OR 2.44, 95% CI 1.39–4.27) than to contacts aged 35–54 years (OR 0.70, 95% CI 0.50–0.98) or ≥55 years (OR 0.39, 95% CI 0.27–0.57). Other factors associated with treatment prescription were household contact with the index case, exposure ≥6 h/day to the index case, tobacco use, and a TST induration diameter measuring 16–20.9 mm or 21–30.9 mm. The factors associated with no treatment prescription were workplace contact with the index case and BCG vaccination.

### 3.2. Characteristics of Pulmonary TB Index Cases

Table 2 summarizes details for the 542 pulmonary TB index cases for whom infected contacts were traced. The only factors associated with treatment prescription were acid-fast-stain (AFS) positivity (overall: OR 2.16, 95% CI 1.11–4.18; ≥6 years: OR 2.10, 95% CI 1.08–4.08) and HIV positivity (overall: OR 3.52, 95% CI 1.35–9.20; ≥6 years: OR 3.36, 95% CI 1.29–8.80).

### 3.3. Factors Associated with Treatment Prescription to Infected Contacts

**Figure 2:**
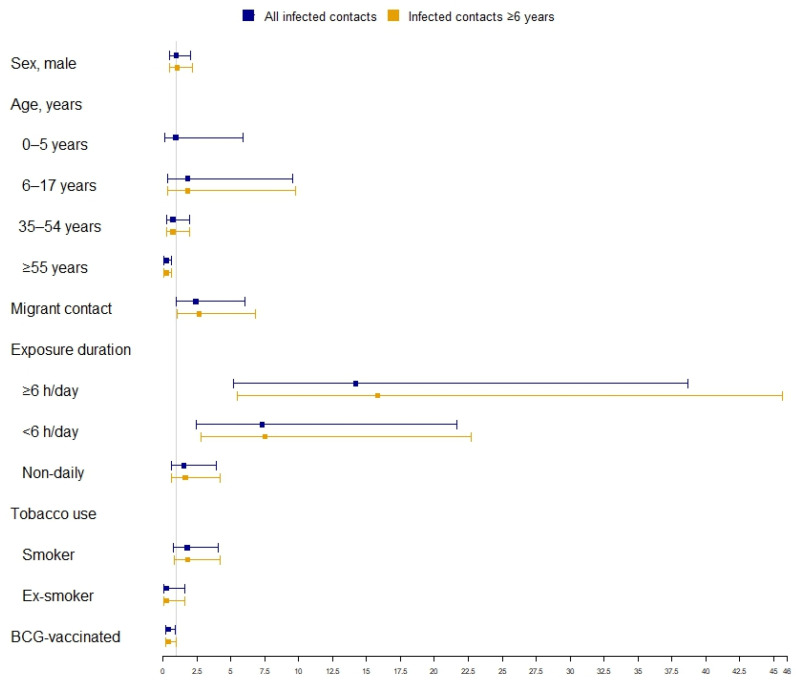
Factors associated with treatment prescription to infected contacts of pulmonary TB index cases.

Figure 2 reports the multivariate analysis results regarding the associations between the studied variables and LTBI treatment prescription. The factors associated with LTBI treatment prescription were exposure of ≥6 h/day (overall: aOR 14.20; 95% CI 5.22–38.66; ≥6 years: aOR 15.83, 95% CI 5.49–45.64), <6 h/day (overall: aOR 7.32, 95% CI 2.48–21.64; ≥6 years: aOR 7.52, 95% CI 2.79–22.74), and, only for contacts aged ≥6 years, migrant status (aOR 2.66, 95% CI 1.04–6.80). The factors associated with no treatment prescription were age ≥55 years (aOR 0.22, 95% CI 0.08–0.64) and BCG vaccination (overall: aOR 0.38, 95% CI 0.16–0.90; ≥6 years: aOR 0.40, 95% CI 0.17–0.98).

## 4. Discussion

LTBI treatment prescription after a medical evaluation of the contacts exposed to active TB cases is crucial to TB prevention [15,16]. In our study, LTBI treatment was prescribed to 80.6% of pulmonary TB contacts (79.9% of contacts ≥6 years), a proportion similar to the 80.2% reported by Sullivan et al. [17] in a study carried out at a community health center catering to a large non-US-born population.

Age was an important factor in decisions regarding LTBI treatment prescription. The bivariate analysis revealed that treatment was more frequently prescribed to children and adolescents (aged 0–5 and 6–17 years) and less frequently prescribed to individuals aged 35 years and older, a result that corroborates that reported for Australia by Dobler et al. [18]. In one US study by Tang et al. [19] carried out at a community health center, LTBI treatment was most frequently prescribed to contacts aged 6–17 years and less frequently to contacts older than 50 years. However, our multivariate analysis pointed to a significant association between no treatment prescription only for the contact group aged ≥55 years.

LTBI treatment prescription was also associated with migrant contacts aged ≥6 years. LTBI treatment decisions regarding migrants from countries with high TB incidence continues to be challenging, as the rate of active TB development over the long term appears to be higher in migrants with detected TB infection [20,21]. While Tang et al. [19] reported no association between migrant status and LTBI treatment prescription, our results suggesting that Catalan physicians take migrant status into account when prescribing treatment corroborates findings for a study carried out among new migrants in Montreal [22].

A noteworthy result of our study was the strong association found for exposure duration to the pulmonary TB index case, not only for contacts exposed for ≥6 h/day, but also for contacts exposed for <6 h/day. Dobler et al. [18] reported that non-household contacts were less frequently prescribed treatment than household contacts, although no information was provided on the exposure duration for household contacts. In our study, we found that LTBI treatment prescription overall and to contacts aged ≥6 years exposed to the TB index case for <6 h/day was around seven-fold greater than for sporadic exposure.

In assessing risks and priorities in contact studies, one of the factors considered is exposure to the index case, with an exposure of ≥6 h/day considered the highest priority for treatment prescription [14]. In our study, LTBI contacts exposed for ≥6 h/day showed the highest association with treatment prescription (around 14 and 15 times greater overall and for contacts aged ≥6 years, respectively, than for sporadic exposure)—which seems entirely logical given the high exposure level. However, the fact that the exposure of <6 h/day was also highly associated with treatment prescription would point to accurate risk assessment in clinical practice in Catalonia. Once treatment is prescribed, it is also important to monitor adherence in contacts exposed for <6 h/day, bearing in mind that, as reported by Gullón et al. [23] for Spain, an exposure level of <6 h/day is significantly related to a failure to initiate treatment.

While tobacco use was more frequently associated with LTBI treatment prescription than non-tobacco use in our bivariate analysis, the association was not statistically significant in the multivariate analysis. Other authors have reported similar findings, i.e., no association [24], or bivariate analysis association but no adjusted analysis association [18]. In their study in Catalonia, Godoy et al. [25] reported an association between smoking and LTBI, but reported no data on the association with treatment prescription. As pointed out by several authors [26,27,28], once close contacts have been identified, the data on both TB risk and treatment non-adherence risk need to be recorded so that the treatment of infected contacts can be prioritized.

In contrast to the Richards et al. [22] finding (for Canada) of no association between BCG vaccination in contacts and LTBI treatment prescription, we found that BCG-vaccinated contacts were less likely to be prescribed LTBI treatment than non-vaccinated contacts. In contrast to the recommendations for the management of TB contacts with a history of BCG vaccination [7,14], 36% of vaccinated contacts in our study had not undergone an IGRA test.

Although more research is needed on this point, a possible explanation could be physician doubts about TB diagnosis in BCG-vaccinated contacts. As suggested by several authors, knowledge of physician attitudes to TB prevention may be useful in advancing the elimination of the disease [29,30].

An exhaustive literature search retrieved the articles cited in the discussion section, but identified no studies reporting data for other Spanish regions. TB treatment prescription guidelines [14], as applied in Catalonia and in all other regions, follow Spanish TB Prevention and Control Plan [31] standards.

The main limitation of our study was missing data, largely attributable to the fact that the study was partially carried out during the first 18 months of the COVID-19 pandemic. It is likely that the corresponding impact on human resources meant that the recovery of a high proportion of the missing data by Catalan Epidemiological Surveillance Network staff was affected. Nonetheless, the large volume of data collected and analyzed would suggest that our results are consistent. A strength of our study is its population-based nature, as the whole population of Catalonia was covered for the 30-month period from 1 January 2019 to 30 June 2021 (12 months before and 18 months during the COVID-19 pandemic).

## 5. Conclusions

Our data would suggest that the information on the exposure duration to pulmonary TB index cases is crucial to LTBI treatment prescription, and that this information is important not only for exposure of ≥6 h/day, but also for lower daily exposure levels. IGRA testing should be carried out in all BCG-vaccinated contacts. Further research is needed on treatment prescription and non-prescription to contacts aged ≥55 years. LTBI testing and treatment of pulmonary TB index case contacts, as a cornerstone of the WHO End TB Strategy, is an essential component in worldwide TB prevention and control and should be practiced by all TB control teams.

Recommendations:

- All pulmonary TB index case contacts should be routinely tested for LTBI and treated as necessary by TB control teams.
- IGRA testing should be carried out in all BCG-vaccinated contacts.
- Information on the exposure duration to pulmonary TB index cases is crucial to LTBI treatment prescription.
- Further research is needed on treatment prescription to contacts aged ≥55 years.

## Figures and Tables

**Table 1:** Characteristics of infected contacts of pulmonary TB index cases according to treatment prescription.

	All infected Contacts Treatment Prescribed			Infected Contacts 0–5 Years Treatment Prescribed			Infected Contacts ≥6 Years Treatment Prescribed		
	Yes	No	aOR (95% CI)	Yes	No	aOR (95% CI)	Yes	No	aOR (95% CI)
Sex									
Male	675	162	1.00 (0.77–1.30)	32	2	0.59 (0.05–6.90)	637	160	1.01 (0.78–1.31)
Female	523	126	Reference	27	1	Reference	494	125	Reference
m.d.	1	0							
Age, years									
0–5	59	3	3.86 (1.17–12.70)	59	3				
6–17	211	17	2.44 (1.39–4.27)				211	17	2.44 (1.39–4.27)
18–35	326	64	Reference				326	64	Reference
35–54	426	120	0.70 (0.50–0.98)				426	120	0.70 (0.50–0.98)
≥55	168	84	0.39 (0.27–0.57)				168	84	0.39 (0.27–0.57)
m.d.	9	0		0	0		0	0	
Migrant									
Yes	505	137	1.06 (0.82–1.39)	10	1	0.42 (0.02–7.34)	489	136	1.08 (0.83–1.42)
No	496	143	Reference	24	1	Reference	471	142	Reference
m.d.	198	8		25	1		171	7	
Contact converter									
Yes	128	16	1.07 (0.61–1.85)	81	1	0.18 (0.01–3.21)	120	15	1.12 (0.63–1.98)
No	946	126	Reference	44	1	Reference	893	125	Reference
m.d.	125	146		7	1		118	145	
Household									
Yes	658	109	2.25 (1.72–2.93)	40	1	2.11 (0.12–35.50)	611	108	2.18 (1.67–2.86)
No	465	173	Reference	19	1	Reference	446	172	Reference
m.d.	76	6		0	1		74	5	
Setting									
Household	793	138	0.86 (0.38–1.93)	53	2	-	734	139	0.77 (0.32–1.85)
School	92	22	0.62 (0.25–1.56)	2	0	-	90	22	0.58 (0.22–1.55)
Workplace	262	121	0.32 (0.14–0.73)	0	0	-	261	121	0.31 (0.13–0.74)
Recreational	47	7	Reference	4	1	Reference	42	6	Reference
m.d.	5	0		0	0		4	0	
Exposure duration									
≥6 h/day	717	106	2.59 (1.70–3.94)	43	1	7.17 (0.39–130.31)	670	105	2.55 (1.66–3.92)
<6 h/day	223	57	1.50 (0.94–2.39)	7	1	1.17 (0.06–22.94)	213	56	1.52 (0.94–2.45)
Non-daily	131	81	0.62 (0.39–0.98)	3	0	-	128	81	0.63 (0.40–1.01)
Sporadic	102	39	Reference	6	1	Reference	95	38	Reference
m.d.	26	5		0	0		25	5	
Tobacco use									
Smoker	337	40	1.70 (1.16–2.50)	0	0		337	40	1.86 (1.26–2.74)
Ex-smoker	23	6	0.77 (0.31–1.94)	0	0		23	6	0.85 (0.34–2.13)
Non-smoker	570	115	Reference	59	3	Reference	507	112	Reference
m.d.	269	127		0	0		264	127	
Alcohol use									
Yes	42	8	0.93 (0.43–2.02)	0	0		42	8	0.96 (0.44–2.09)
No	859	152	Reference	45	3	Reference	812	149	Reference
m.d.	298	128		14	0		277	128	
BCG-vaccinated									
Yes	330	113	0.43 (0.31–0.59)	11	1	0.56 (0.05–6.82)	318	112	0.45 (0.33–0.63)
No	541	80	Reference	39	2	Reference	499	78	Reference
m.d.	328	95		9	0		314	95	
HIV status									
Positive	8	1	1.10 (0.14–8.93)	0	0		8	1	1.13 (0.14–9.13)
Negative	602	83	Reference	25	2	Reference	575	81	Reference
m.d.	589	204		34	1		548	203	
Immunosuppressed									
Yes	6	2	0.39 (0.08–1.98)	0	0		6	2	0.40 (0.08–2.03)
No	725	95	Reference	32	2	Reference	691	93	Reference
m.d.	468	191		27	1		434	190	
TST, mm									
0–4.9	81	29	Reference	3	0	Reference	78	29	Reference
5–9.9	72	59	0.44 (0.25–0.75)	1	0		71	59	0.44 (0.25–0.77)
10–15.9	275	91	1.08 (0.67–1.76)	17	1		257	90	1.06 (0.65–1.73)
16–20.9	271	41	2.37 (1.38–4.05)	11	0		259	41	2.35 (1.37–4.03)
21–30.9	163	21	2.78 (1.49–5.17)	2	0		159	21	2.82 (1.51–5.25)
≥31	41	16	0.92 (0.45–1.88)	0	1		41	15	1.02 (0.49–2.11)
m.d.	296	31		25	1		266	30	

aOR: adjusted odds ratio; BCG: bacillus Calmette–Guerin; CI: confidence interval; HIV: human immunodeficiency virus; TST: tuberculin skin test; m.d.: missing data.

**Table 2:** Characteristics of pulmonary TB index cases whose infected contacts were analyzed according to treatment prescription.

	Index Cases of All Infected Contacts Treatment Prescribed			Index Cases of Infected Contacts 0–5 Years Treatment Prescribed			Index Cases of Infected Contacts ≥6 Years Treatment Prescribed		
	Yes	No	aOR (95% CI)	Yes	No	aOR (95% CI)	Yes	No	aOR (95% CI)
Sex									
Male	247	65	1.27 (0.81–1.97)	12	1	1.20 (0.07–21.72)	232	64	1.28 (0.81–2.01)
Female	126	42	Reference	10	1	Reference	116	41	Reference
m.d.	60	2		1	0		59	2	
Age, years									
0–5	3	1	0.71 (0.07–7.04)	0	0	-	3	1	0.76 (0.08–7.50)
6–17	34	10	0.81 (0.37–1.78)	1	1	-	32	9	0.90 (0.40–2.04)
18–35	164	39	Reference	9	0	Reference	154	39	Reference
35–54	145	31	1.11 (0.66–1.87)	10	0	-	135	31	1.10 (0.65–1.86)
≥55	84	28	0.71 (0.41–1.24)	3	1	-	80	27	0.75 (0.43–1.31)
m.d.	3	0		0	0		3	0	
Migrant									
Yes	234	53	1.29 (0.84–1.98)	13	2	-	220	51	1.34 (0.87–2.08)
No	181	53	Reference	10	0	Reference	170	53	Reference
m.d.	18	3		0	0		17	3	
Chest X-ray									
Cavity	174	34	2.19 (0.78–6.11)	12	1	-	161	33	2.09 (0.75–5.84)
No cavity	212	65	1.40 (0.51–3.78)	11	1	-	200	64	1.34 (0.49–3.63)
Normal	8	1	Reference	0	0	Reference	8	1	Reference
Not done	14	6	3.43 (0.35–33.80)	0	0		14	6	3.43 (0.35–33.80)
m.d.	25	3		0	0		24	3	
Bacteriology									
AFS-positive	237	40	2.16 (1.11–4.18)	16	0	-	220	40	2.18 (1.08–4.08)
Culture-positive	125	48	0.95 (0.49–1.84)	5	2	-	120	46	0.99 (0.51–1.94)
Negative	44	16	Reference	2	0	Reference	42	16	Reference
Not done	6	1	2.18 (0.24–19.55)	0	0		5	1	1.90 (0.21–17.59)
m.d.	21	4		0	0		20	4	
Social vulnerability									
Yes	32	5	1.66 (0.63–4.36)	3	0	-	29	5	1.56 (0.59–4.14)
No	401	104	Reference	20	2	Reference	378	102	Reference
m.d.	0	0		0	0		0	0	
Tobacco use									
Yes	162	35	1.16 (0.71–1.86)	6	0	-	155	35	1.16 (0.72–1.87)
No	212	53	Reference	16	2	Reference	195	51	Reference
m.d.	59	21		1	0		57	21	
Alcohol use									
Yes	54	11	1.18 (0.59–2.37)	3	0	-	51	11	1.16 (0.58–2.34)
No	307	74	Reference	19	2	Reference	287	72	Reference
m.d.	72	24		1	0		69	24	
Diabetes									
Yes	32	8	0.91 (0.40–2.06)	2	0	-	30	8	0.89 (0.39–2.02)
No	316	72	Reference	20	2	Reference	295	70	Reference
m.d.	85	29		1	0		82	29	
HIV status									
Positive	10	8	3.52 (1.35–9.20)	0	0	-	10	8	3.36 (1.29–8.80)
Negative	352	80	Reference	22	2	Reference	328	78	Reference
m.d.	71	21		1	0		69	21	
IDU									
Yes	7	4	0.39 (0.11–1.36)	1	0	-	6	4	0.35 (0.10–1.26)
No	343	76	Reference	21	2	Reference	321	74	Reference
m.d.	83	29		1	0		80	29	

AFS: acid-fast stain; aOR: adjusted odds ratio; CI: confidence interval; HIV: human immunodeficiency virus; IDU: injecting drug user; m.d.: missing data

## Data Availability

The datasets used and/or analyzed during the current study are available from the corresponding author upon reasonable request.
